# On the Structural, Thermal, Micromechanical and Tribological Characterizations of Cu-Filled Acrylonitrile Butadiene Styrene Micro-Composites

**DOI:** 10.3390/ma16196428

**Published:** 2023-09-27

**Authors:** Mabrouka Akrout, Basma Ben Difallah, Mohamed Kharrat, Maher Dammak, António Pereira, Filipe J. Oliveira, Isabel Duarte

**Affiliations:** 1 Laboratory of Electromechanical Systems, National School of Engineers of Sfax, University of Sfax, Soukra Road, Km 3.5, PO Box 1173, Sfax 3038, Tunisia; mabrouka.akrout55@gmail.com (M.A.); basma.bendhaifallah@ipeis.usf.tn (B.B.D.); maher.dammak@ipeis.rnu.tn (M.D.); 2 Department of Mechanical Engineering, Centre for Mechanical Technology and Automation (TEMA), University of Aveiro, 3810-193 Aveiro, Portugal; abastos@ua.pt (A.P.); isabel.duarte@ua.pt (I.D.); 3 LASI—Intelligent Systems Associate Laboratory, 4800-058 Guimarães, Portugal; 4 CICECO, Department of Materials Engineering and Ceramics, University of Aveiro, 3810-193 Aveiro, Portugal; filipe@ua.pt

**Keywords:** ABS, Cu, micro-composite, microstructure, thermal, nanoindentation, tribology

## Abstract

The purpose of this work was to investigate the structural, thermal, micromechanical and tribological properties of novel polymer/metal composite materials for bearing applications. Copper (Cu)-filled Acrylonitrile Butadiene Styrene (ABS) composites were mixed in a laboratory scale by an internal mixer with two blade impellers, and then injection-molded. Neat ABS, ABS+5wt% Cu, ABS+10wt% Cu, and ABS+15wt% Cu were the four materials that were tested. The dispersion of Cu particles in the ABS matrix was investigated using Scanning Electron Microscopy (SEM) and a micro-tomography scan. The filler particles have a uniform distribution in the matrix, according to the observations. The incorporation of Cu filler also refined an increase in the glass transition temperature from Differential Scanning Calorimetry (DSC) and less intensity in the amorphous phase by X-ray diffraction (XRD). Nanoindentation tests were carried out to characterize the micro-mechanical behavior of the composites. Friction and wear analysis were also examined using a pin-on-disk tribometer. Compared with neat ABS, all the micro-composites showed much higher indentation hardness, Vickers hardness, and indentation elastic modulus. It was also concluded that the incorporation of Cu filler into ABS simultaneously improved the friction and wear properties of the composites, which contributed to the suitability of the micro-filled composites with hard metallic particles for a wider range of mechanical components for bearing applications.

## 1. Introduction

With regards to their low cost, light weight, ductility, good friction and wear qualities, and easy processability, polymers are increasingly used in the replacement of metal parts in mechanical systems. Polymers can be introduced in many technological applications such as brakes, gears, and electrical appliances [1,2].

If important mechanical loading or contact pressure is needed, the use of polymers may be hindered. Herein comes the role of introducing different fillers to reinforce polymers, and hence, to overcome these restrictions. The shape and size of the fillers (fibers, microparticles, nanoparticles, etc.) as well as their material’s nature (glass, carbon, metal, textiles, etc.) are mostly determined by the desired properties. Interest in the development of polymer composites has advanced quicker than that of metals due to their enhanced required properties in a wide range of mechanical parts [3,4]. Reinforcing a polymer matrix with fibers or fillers can modify the polymer’s tribological, mechanical, and thermal properties [5,6,7,8]. Shang et al. [9] focused on the development and analysis of composites by combining Polyether Ether Ketone (PEEK) and Multi-Walled Carbon Nanotubes (MWCNTs) to harness their synergies, enhancing mechanical strength and tribological properties. For instance, the addition of several types of fillers to a PTFE matrix reduced wear significantly; Charfi et al. [3] reported that the PTFE+bronze mix provided good wear resistance.

Metal particles have been used to improve the electrical, thermal, and mechanical properties of polymers [10,11]. Tavman [12] investigated the thermal and mechanical characteristics of polyethylene composites loaded with copper powder. It was found that copper particles enhanced the composites’ thermal conductivity and had a detrimental impact on their mechanical qualities. Few studies discussed the effect of metal particles on the tribological properties of polymers, including those by Rajesh and Bijwe [13] and Trabelsi et al. [4]. According to the findings of Brostow et al. [14], the incorporation of Al, Ag or Ni micro-sized powders to a polymeric matrix such as low-density polyethylene (LDPE) or thermoplastic elastomer (TPE) can produce composites with improved mechanical and tribological characteristics, making them appropriate for a variety of industrial applications. Valuable insights into the wear behavior of epoxy composites filled with copper, aluminum, and iron metallic particles and scratched by a steel indenter under the influence of a magnetic field were provided by El-Zahraa et al. [15]. The authors concluded that the wear response of the different composites was affected by the presence and the intensity of the magnetic field, as well as the nature of the metallic filler. Trabelsi et al. [4] reported that the commercial PTFE+40wt% Bronze composite used for guide rings enables the best tribological abilities in comparison with commercial pure PTFE and other composites provided commercially. In the work of Uflyand et al. [16], quasicrystalline Al65Cu22Fe13 alloy (QC) incorporated into a linear low-density polyethylene (LLDPE) thermoplastic matrix enhanced the strength properties of the composite materials. According to their research work, the composite filled with 1wt% QC nanoparticles showed a 57% reduction in wear compared to the pure LLDPE.

ABS is an amorphous thermoplastic polymer which is well-known for advanced composites due to its significant mechanical strength, toughness, and chemical resistance. In the present work, composites based on an ABS thermoplastic polymer loaded with various weight fractions of micro-sized copper powder were developed. The goal was to investigate the effect of the presence and amount of copper on the microstructural, thermal, micromechanical and tribological properties of ABS/Cu micro-composites. Considering the hopeful complementary characteristics between the thermoplastic polymer and the hard metallic filler, it is hoped that the combination of the two materials simultaneously improve the micro-mechanical and the tribological properties established in ABS, which would promote their practical applications in large-scale production and in a sustainable way.

## 2. Materials and Methods

### 2.1. Materials

ABS pellets (1.04 g/cm^3^ density, 7.6 g/10 min IF) were supplied by Iran’s Ghaed Bassir Petrochemical Co. (Golpayegan, Iran). Copper powder with an average particle size of 75 µm, a density of 8.94 g/cm^3^ and a melting point of 1080 °C was used. The copper material was purchased from Sigma Aldrich (St. Louis, MO, USA). The ABS matrix is an impact-resistant amorphous thermoplastic with high rigidity and dimension stability. Cu powder was incorporated into the polymer for the purpose of increasing its stiffness and to produce a strengthening effect. Before processing, ABS pellets were vacuum-dried for at least 4 h at 80 °C.

### 2.2. Preparation of Micro-Composites

**Figure 1:**
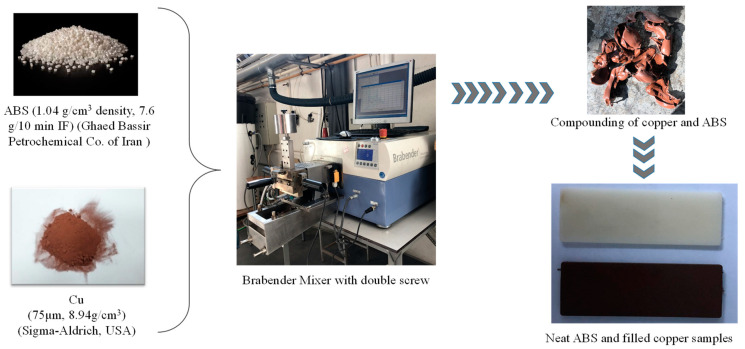
Experimental methodology for the development of ABS/Cu micro-composites.

Figure 1 illustrates the experimental procedure used to develop the different micro-composites. The composites were formed by blending the polymer and copper powder ratios in a Brabender Plastograph internal mixer with two blade impellers (Brabender, Duisburg, Germany) shown in Figure 1. The two materials were mixed for 8 min at 175 °C with a speed of 75 rpm in a 20 cm^3^ mixing chamber. The resulting blends with different copper content were pelletized and then injection-molded using a mini-injection molding machine type HAAKE Mini Jet (Thermo Scientific, Waltham, MA, USA). The cylinder temperature, mold temperature, and injection pressure were 230 °C, 110 °C, and 600 bars, respectively. The composites were produced with copper powder fractions ranging from 0% to 5%, 10%, and 15% by weight.

### 2.3. Characterizations

One specimen from each type of composites was requested in a Discovery DSC 250 Differential Scanning Calorimeter from TA Instruments (New Castle, DE, USA). The equipment was used to measure the glass transition temperature (Tg) and heat flow for the different ABS/Cu micro-composites. In this investigation, the different specimens were extruded onto aluminum pans with sample weights ranging from 13 to 16 mg. The composites were heated to 250 °C at a heating rate of 10 °C/min to obtain acceptable resolution for both ABS and its composites.

To determine the phase composition, XRD measurements were performed at room temperature using a Bruker D8-Advance diffractometer (Bruker, Billerica, MA, USA) with Cu Kα radiation (λ = 1.540593Å) under a tube voltage of 40 kV. The angular range was 5–80° (2θ) and the scanning speed was 3°/min using the step scanning mode (0.02 step^−1^) with a counting time of 2 s step^−1^.

Different morphological characterizations were employed to examine the composites’ microstructures. The dispersion of Cu copper particles in the ABS matrix was investigated using back-scattered electrons from an SEM, Hitachi microscope Su-70 Schottky Field Emission, Toronto, ON, Canada (at 15 kV). The fracture surfaces of ABS micro-composites at different weight fractions of Cu filler were examined. For this purpose, the samples were frozen with liquid nitrogen to a temperature of −200 °C and then broken.

Tomographical reconstructions allowed us to obtain three-dimensional information about the composites’ internal microstructures. An X-ray microcomputed tomography (µCT) machine (SkyScan 1275, Bruker, Kontich, Belgium) was used with penetrative X-rays of 100 kV and 100 µA, 1 mm Cu filter. A high-resolution mode was also employed with a pixel size of 12 µm, a rotation step of 0.2° until 360° rotation, a frame averaging of 3 and an exposure time of 200 ms and 150 ms, respectively. For 3D reconstruction and visualization, the software NRecon, DataViewer and CTVox (Bruker, Kontich, Belgium, 2016) were used.

The application of nanoindentation in the characterization of inhomogeneous thermoplastic composites is used in a fast-growing research area to analyze the materials composed of discrete regions with different properties. The nanoindentation test (TTX-NHT Nanoindentation Tester, CSM Instruments, Needham Heights, MA, USA) was conducted to investigate the micromechanical properties of our ABS/Cu composites. The tester was attached to a Berkovich diamond indenter with a 20 nm edge radius facing 65.30° from the vertical axis. Low depth regimes were used in indenting the thermoplastic composites for precise determination of the indentation characteristics. Different loads ranging from 5 mN to 150 mN were applied at various surface regions of each sample. A minimum of five samples per type of material were tested and the average values of hardness and modulus were obtained with standard deviations. The approach speed, dwell period, and loading/unloading speed were all held constant at 2000 nm/min, 10 s, and 200 mN/min, respectively. During the nanoindentation test, the parameters measured include the indentation depth (h) and the loading force (P). An unloading sequence follows the test and provides useful information about the stiffness of the contact (dP/dh), which is necessary to measure the changes in hardness or modulus with the penetration depth.

**Figure 2:**
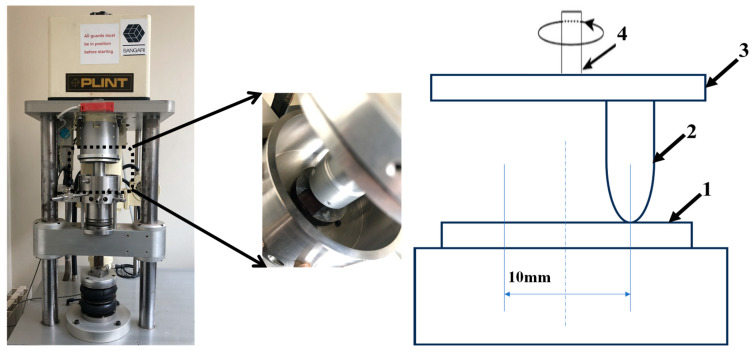
Setup for tribological tests. (**Left**) Pin-on-disk tribometer PLINT (TE-92 device). (**Right**) Schematic configuration of the tribological test [16]: 1—ABS sample, 2—steel pins, 3—holder, 4—axis of rotation.

Frictional tests were also conducted using a pin-on-disk tribometer (TE-92 device, Phoenix Tribology Ltd., Newbury, UK) at room temperature under a normal load of 50 N (Figure 2). The tribometer was described in a previous research work [16]. A 7.8 mm diameter steel pin (115CrV3, Virgamet, Koło, Poland) was rotated at a sliding speed of 75 rpm against a rectangular 50 × 20 × 2 mm^3^ composite sample. The sliding track was 10 mm in diameter. The width and cross-section of the wear track for each sample tested were evaluated on a profile established using the Sensofar profilometer (Sensofar, Barcelona, Spain) after being treated by Mountains Lab Premium 9. The wear volume loss was determined as:

V = Π d S(1)

where d is the diameter of sliding track equal to 10 mm and S is the measured cross section of the wear track.

## 3. Results and Discussions

### 3.1. Structural and Morphological Aspects

**Figure 3:**
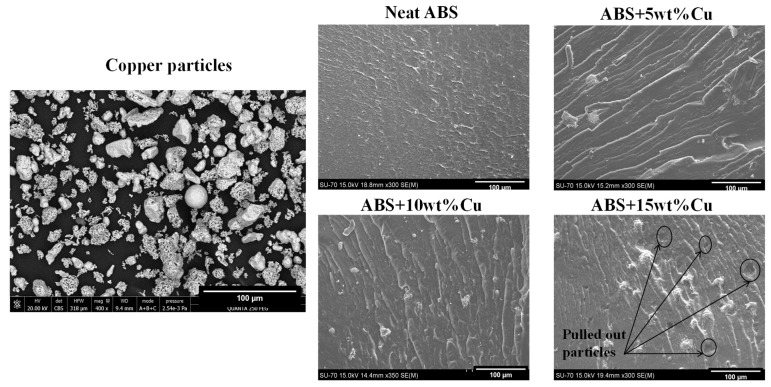
SEM of the copper particles and the cryofractured surfaces of the neat ABS and its micro-composites.

Scanning electron micrographs of cryofractured surfaces in neat ABS, ABS+5wt% Cu, ABS+10wt% Cu, and ABS+15wt% Cu composites and the morphology of pure copper powder (400x) are shown in Figure 3. The SEM image of Cu powder shows that most of the larger particles had a spherical shape while the smaller ones are agglomerated in an irregular shape with many differences in size distribution. SEM results also clearly show that the surface of the neat ABS sample is almost smooth, with straight grooves indicating a brittle fracture mechanism. A more ductile appearance can be observed with the ABS/Cu composites probably due to their high level of impact strength and fracture energy [17,18]. The features also indicate that the interfacial adhesion between the filler and the matrix is relatively poor. Pulled-out particles leading to voids as well as signs of debonding between copper particles and the ABS matrix are clearly distinguishable. The wetting of the metallic filler by the ABS matrix is probably insufficient and a coupling agent may be used to enhance the adhesion between metal particles and matrix. Noticeably, a uniform distribution of copper particles in the ABS matrix for all the considered ABS/Cu composites is seen. According to the findings, the composites had no evident flaws. Homogeneity in the dispersion of copper particles in the ABS polymer matrix can help to improve their mechanical and tribological properties.

**Figure 4:**
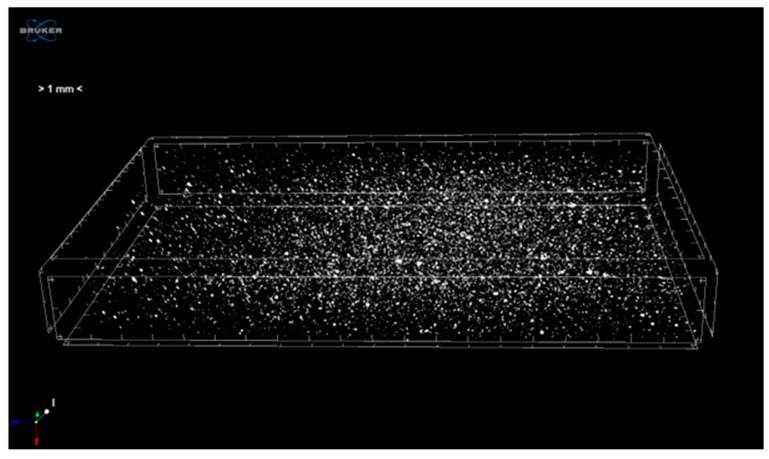
Microtomography visualizations in 3D images of the ABS+10wt% Cu composite.

A three-dimensional (3D) X-ray projection image of the ABS+10wt% Cu composite, developed with the µ-tomography technique, is shown in Figure 4. The 3D microstructure of the composite is obtained through the computational reconstruction of the X-ray projections. Imaging is mainly used to distinguish between the filler and the matrix. The matrix is represented by black color regions, whereas the porosities are represented by regions with a gray color. Similarly, the copper particles are represented by the areas with the maximum clarity (or white color). Occasionally, discrete voids are dispersed throughout the ABS matrix. The low density of pores indicates that the injection process parameters are optimized.

The three-dimensional reconstructions also reveal good particle dispersion inside the polymer matrix despite the existence of a few copper particle agglomerates. The last result is mainly due to the mixing step of the composites offered by the Brabender Plastograph internal mixer. The SEM results of the highly dispersed morphology of Cu filler throughout the ABS matrix are confirmed by the tomographic imaging.

**Figure 5:**
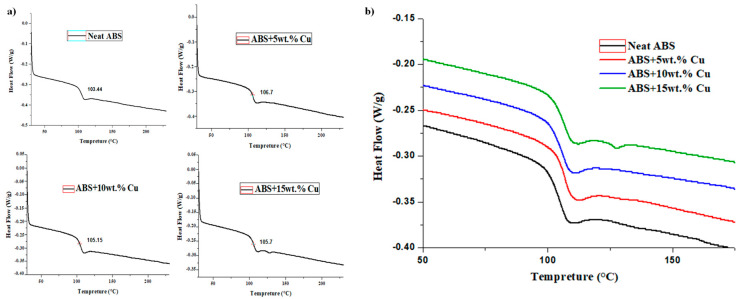
(**a**) DSC analysis of ABS/Cu micro-composite samples at different Cu loading. (**b**) Superposition of the DSC curves of the ABS/Cu micro-composites.

The principal goal of the DSC test was to determine the influence of copper particles incorporated into ABS during the thermal processing stages, including the use of the Brabender Plastograph internal mixer and injection-molding process on the Tg temperature values. Table 1 displays the findings for ABS pure material and ABS/Cu composites. From the results acquired, the increment of copper filled in the ABS matrix has a slight influence on the glass transition temperature Tg of the composites. It has been noticed that the Tg value rises in the copper-filled ABS matrix when it is compared to the neat polymer. Particularly, the glass transition temperature Tg increased from 103.44 °C for pure ABS with a heat flow of −0.339 W/g to 106.7 °C for ABS+5wt% Cu with −0.322 W/g. DSC thermograms in Figure 5a,b show the variations in the glass transition temperatures in neat ABS and ABS/Cu micro-composites. Generally, the onset of glass transition corresponds to the first segments’ vibrations of an entangled polymer chain which is a general phenomenon in any semi-crystalline or amorphous polymer. The improvement in glass transition temperature can be related to the reduced onset of segmental mobility. This suggests that, at 5wt% loading, the copper particles are deeply introduced into the ABS thermoplastic chains forming a network like a structure, thereby restricting the segmental motions of the ABS chains and improving the glass transition temperature [19,20].

**Figure 6:**
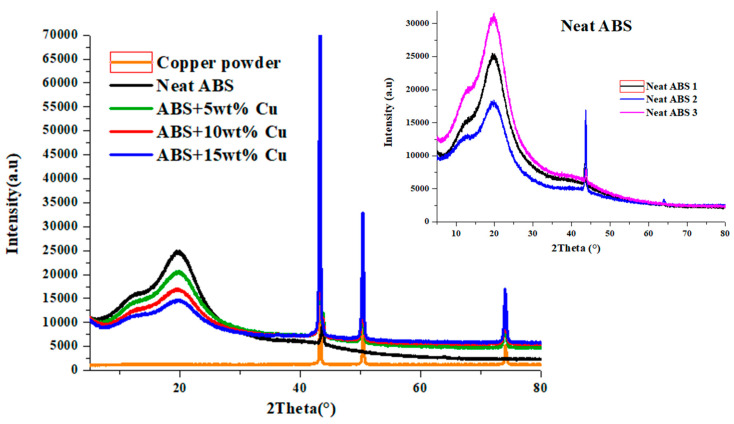
X-ray diffractograms of neat ABS and its micro-composites.

XRD analysis was performed to characterize the changes in the copolymer structure caused by the copper addition. Figure 6 displays the background removal from XRD patterns for all four ABS/Cu micro-composites at different Cu loading and the copper powder. The observed patterns in the neat ABS are comparable to those published in the studies of Bandeira et al. [21] and Azerag et al. [22]. The spectra of the four ABS/Cu micro-composites in the figure have two broad major peaks at 13.8° and 19.9° that are characteristic of the amorphous structure. A lower intensity in the amorphous phase was observed with the ABS/Cu micro-composites; apparently, the copper particles were intercalated between the ABS amorphous molecular chains. In addition to the fundamental amorphous phases, a third peak is also seen at 43.6° in neat ABS. However, previous research examining pure ABS missed this peak [21,22]. This proved that the ABS material had seen molecular changes because of the thermal processing stage. The last result can be categorized under the turbostratic band of the disordered carbon substance used in the bulk polymer which caused the peak [23]. The magnitude of the last diffraction peak becomes more noticeable as the copper fraction rises for all ABS/Cu micro-composites. Distinctive copper peaks are also seen at 2θ = 50.38° and at 2θ = 74.04°. Obviously, the XRD peaks of copper are also visible in the ABS/Cu micro-composites. They are heightened and enlarged upon increasing the filler loading in the ABS matrix. This suggests that copper microparticles were not perfectly dispersed throughout the ABS matrix because XRD patterns cannot detect the inherent crystal diffraction of Cu particles in the case of a perfect dispersion [24].

### 3.2. Micromechanical Characterizations

**Figure 7:**
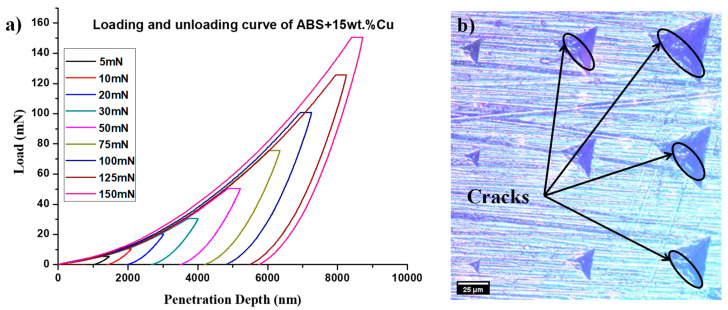
(**a**) Load-penetration depth curve for the ABS+15wt% Cu. (**b**) Optical image of the ABS+15wt% Cu after indentation for the different applied loads.

Consideration of both mechanical stability and tribological qualities, such as friction and wear, is necessary when developing composite materials for sliding contact applications. Particularly, the mechanical characteristics, such as hardness and elastic modulus, of ABS/copper micro-composites have a significant role on their resistance to damage. Contrary to the volumetric characterizations (hardness measurements, traction test, etc.), nanoindentation is an experiment conducted to recover the mechanical properties at the nanometer or sub-micrometer scale. This scale is perfect to characterize the material properties in the top layers of the contact surfaces involved during sliding tests. Figure 7a shows typical load–depth superposed curves recorded in the ABS+15wt% Cu composite with different maximum applied forces ranging from 5 mN to 150 mN. The distance between the indentations was about 50 µm and suitable regions were selected before the tests with the support of microscopy. Test results include correct and consistent values of the elastic modulus which were determined by calculating the slope of the initial linear part of the unloading curves. The indentation hardness can be measured with the maximum load applied and by determining the contact area between the Berkovich indenter and the composite sample at maximum depth after plastic deformation. Figure 7b shows the optical image of the composite surface after indentation. Small contact areas are observed with the lower applied forces, while the larger tracks are obtained with maximum applied forces, ranging from 75 mN to 150 mN. It is important to note that cracks and fractures occurred under a higher indentation load. This process may indicate low interfacial interactions between the copper particles and the ABS matrix which is in good agreement with the observations of the fractured surfaces.

**Figure 8:**
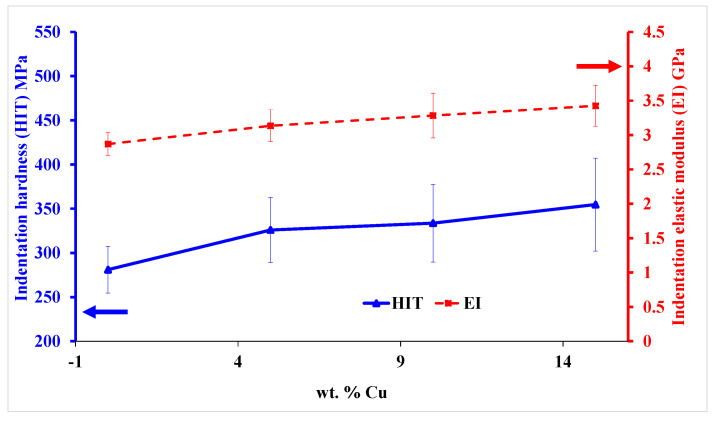
Nanoindentation characterizations of the micro-composites ABS/Cu: the indentation hardness (HIT) with blue color and the indentation elastic modulus (EI) with red color.

As shown in Figure 8, ABS+15wt% Cu is harder than neat ABS, and the indentation hardness (HIT) values range from 281 MPa in the unfilled polymer to 354 MPa with 15wt% copper. With the increase in Cu content, error bars reveal that the dispersion of hardness results become bigger. The fluctuation of hardness values is huge, which may be contributed to the components of the composites that are composed of two kinds of materials. The presence of some particle agglomerations is the main reason leading the phenomenon. Figure 8 also shows the variation in the composite’s elastic modulus (EI); the value rose from 2.868 GPa to 3.426 GPa, in addition to the hardness. The two mechanical parameters continuously increase with the increase in the copper content. Overall, the contact stiffness, modulus, and hardness of the ABS/copper micro-composites were improved by adding copper. The results obtained are consistent with the literature data. It was shown that the introduction of hard metal fillers significantly increases the modulus and hardness of thermoplastic composite materials [14,16].

### 3.3. Friction and Wear Test Results

**Figure 9:**
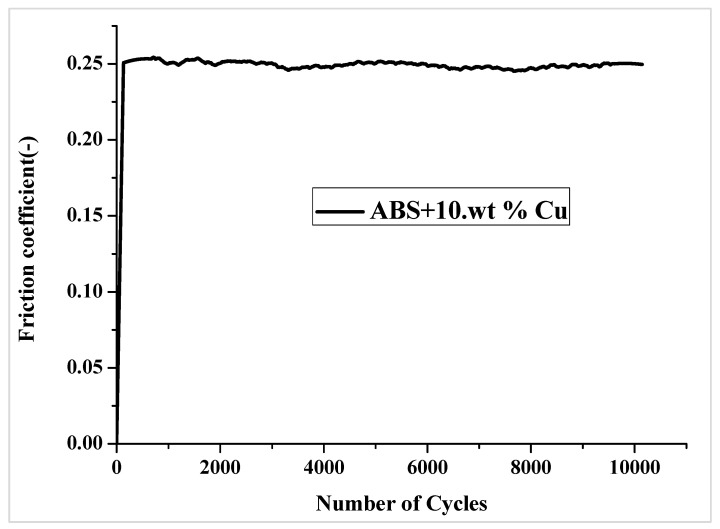
Typical evolution of the friction coefficient versus the number of cycles for ABS+10wt% Cu.

Figure 9 shows a typical curve for the evolution of the friction coefficient as a function of the number of rotating cycles for the ABS+10wt% Cu composite. During the running-in stage, the friction coefficient increased gradually as the number of cycles increased, reaching finally a steady-state period where the friction coefficient remained roughly constant until the end of the test.

**Figure 10:**
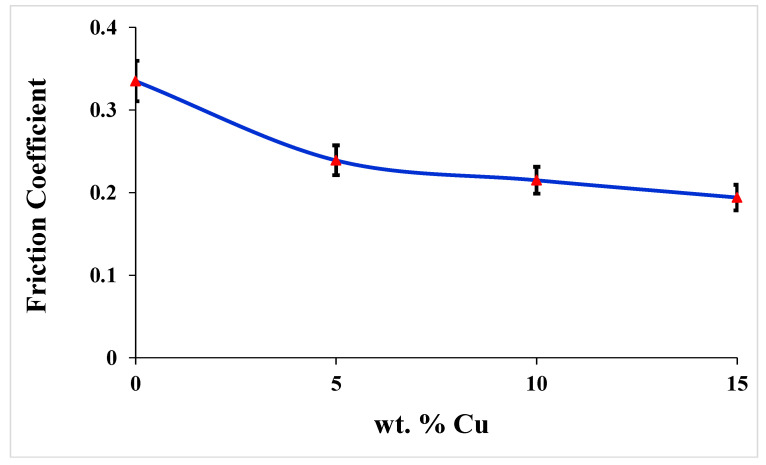
The evolution of the friction coefficient (after 10,000 rotation cycles) as a function of the copper content.

Figure 10 shows the evolution of the stabilized value of the friction coefficient (after 10,000 rotation cycles) as a function of the copper content. With the addition of copper to the ABS matrix, the coefficient of friction is found to be reduced noticeably, and the stabilized value of ABS’s friction coefficient was considerably decreased. Pure ABS exhibits the highest friction coefficient of about 0.356. The ABS+15wt% Cu composite has the lowest friction coefficient of about 0.196. A similar phenomenon was observed by Ben Difallah et al. [25] in the case of graphite (Gr) incorporated into an ABS matrix; it was reported that the best friction properties of ABS were obtained with the highest content of Gr. The increase in indentation hardness and stiffness by adding Cu microparticles have a detrimental impact on lowering the coefficient of friction [26].

**Figure 11:**
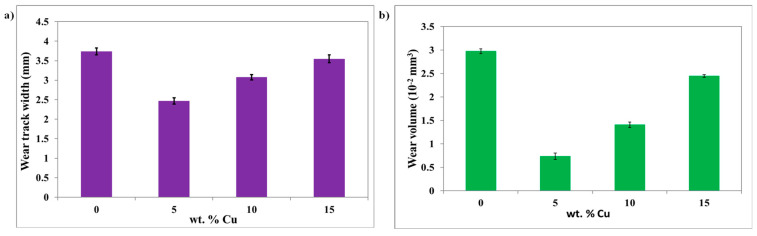
(**a**) Wear track width and (**b**) wear volume loss versus the copper weight fraction after 10,000 cycles for neat ABS and its micro-composites.

The bar chart in Figure 11 shows the wear track width and the volume loss obtained after 10,000 rotating cycles for the considered copper weight fractions. Overall, the wear scar width variation is in accordance with the volume loss results. Both wear properties report the lowest value at 5wt% of copper, after which an increasing tendency is seen when the copper content increases. For instance, the wear scar width value decreases from 3.73 mm for the neat ABS to a minimum value of 2.46 mm at 5wt% Cu, as observed in Figure 11a.

Comparing the pure polymer to the ABS+5wt% Cu composite, the volume loss of ABS dropped from 0.029 mm^3^ to around 0.007 mm^3^. When the copper content is higher than 5wt% Cu, the wear volume tends to rise again. Overall, the wear volume loss values remained lower than that of neat ABS. This may be explained by the generation of a uniform transfer film on the surface of the antagonist with low fractions of copper. The transfer film is formed with better continuity and ductility, which reduced the shearing forces during rotating cycles and protected the two contact surfaces (Steel pin/ABS composites) against wear. Hence, the friction and wear volume loss were significantly reduced [27,28]. Similar tendencies were observed in the wear behavior of POM/Cu micro-composites [27]. The wear rate decreased significantly at 3wt% Cu; a further increase in the copper weight fraction regressed the anti-wear abilities of the composites. It is also worth mentioning that the incorporation of copper microparticles significantly improved the ABS’s wear resistance. The composite with 5wt% copper experiences the best anti-wear abilities. In the study of Bahadur and Gong [29], the authors used three copper compounds (CuS, CuO, CuF_2_) to enhance the tribological properties of a PEEK matrix. Many differences in the evolutions of the coefficient of friction and those of the volume loss were recorded. Different tendencies were observed as a function of the sliding distance as well as the filler nature and loading. The transfer films and their bonding to the counterpart were studied by SEM, XPS, and spectrometry. It has been argued that the reduction of wear depended on the filler’s ability to form a thin and uniform transfer film which is strongly bonded to the antagonist. In the present study, it is found that the evolution trend of the friction coefficient is different from the wear characteristics’ tendency. The wear volume loss increases after incorporating 5wt% of copper into the ABS matrix while the friction coefficient steadily decreased with the copper content. This can be explained by the excessive number of detached copper particles from the composite surface at a higher filler proportion as the adhesion of copper to the ABS matrix is poor. The voids due to particle pull-out are quantified with the wear volume loss, while the frictional performances are steadily enhanced due to the solid lubrication action of copper particles covering the composite surface.

**Figure 12:**
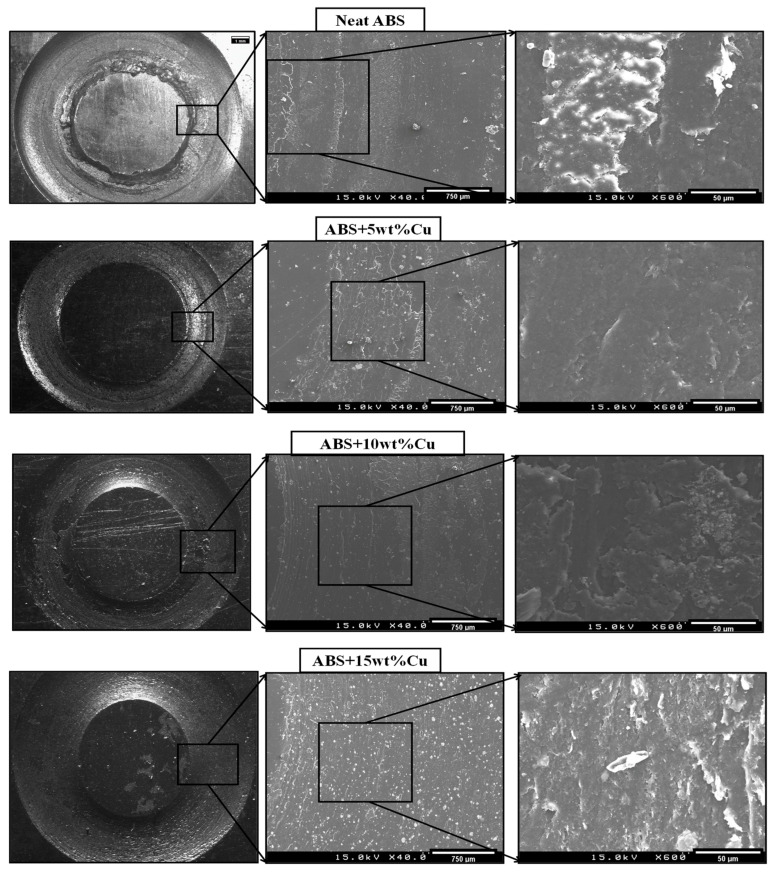
SEM micrographs of the worn surfaces after 10,000 sliding cycles for neat ABS and its micro-composites.

**Figure 13:**
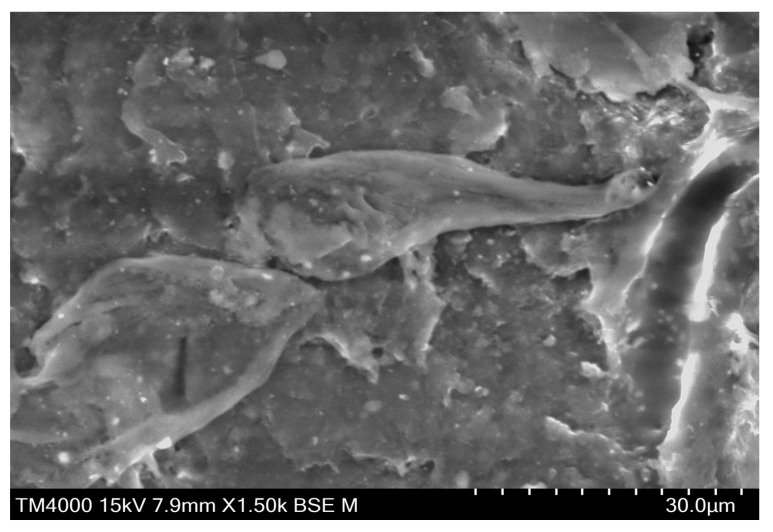
SEM image of the wear debris of the ABS+5wt% Cu micro-composite.

Based on the SEM micrographs, numerous investigations were made to better understand the wear evolutions of polymer composites. The various wear mechanisms of polymer composites can be broadly divided into two groups: the cohesive wear, which includes most mechanical wear processes like abrasion, fatigue, and fretting; and the interfacial wear processes including transfer wear and chemical or corrosive wear [30]. SEM analysis of the worn surface of the ABS/Cu micro-composites was performed to better understand their wear processes when sliding over the steel pin. The SEM images of the worn surface of the neat ABS and ABS/Cu micro-composites after 10,000 sliding cycles were shown in Figure 12. As may be seen, there are numerous apparent traces of ploughs throughout the sliding direction in the worn surface of neat ABS. Hard asperities plowed into the worn surface of neat ABS, causing plastic deformations and indicating that severe adhesive damages were responsible for the material’s wear process. The severity of the wear process was attenuated interestingly on the worn surface of ABS+5wt% Cu. Less ploughs with a little number of wear particles were seen compared to neat ABS. The surface of the composite was clearly smooth, showing a mild adhesive wear process. At higher magnification, the morphology of wear debris changed into thin flakes which may include hard Cu particles and act as solid lubricants. Wear particles may be integrated on the surface of the antagonist as a thin layer restraining the direct contact between the antagonist and the composite sample. As the copper concentration rises, the observations show more wear debris on the worn surfaces of ABS-Cu micro-composites. Ploughing and light abrasion marks are observable in the surface of ABS+10wt% Cu. This phenomenon is more pronounced in the case of the composite with 15wt% Cu. Abrasive scratches and an abundant number of wear debris are the signs of a severe abrasive wear mode. This could be because of the copper particles’ poor compatibility with ABS and the copper particles’ tendency to aggregate in the ABS matrix at high copper contents. As a result, wear debris were quicker to grow and remove out under conditions of prolonged stretching, shearing, and increased temperatures caused by friction during sliding, which reduced wear resistance [27]. Figure 13 displays an SEM image of the ABS+5wt% Cu composite wear debris at higher magnifications. It is evident that the wear debris were irregular in shape such as rolling and lamellar morphology, and their molten surfaces and uneven edges indicated that they had undergone plastic stretching and melting [31].

## 4. Conclusions

Micro-composite materials were prepared on the basis of ABS thermoplastic matrix reinforced with 5, 10 and 15wt% of copper filler. The cryofractured surfaces of ABS/Cu micro-composites were studied using SEM. The observations revealed a uniform dispersion of Cu powder throughout the ABS matrix, which was confirmed by the microtomographic images. The fractured surfaces of the composites also showed a relatively poor adhesion between the ABS matrix and the metal particles. Few particle agglomerations in the micro-composites were also noticed, and this result was confirmed by the increase in the peaks’ intensity as shown in the XRD patterns of our ABS/Cu composites. The thermal properties of ABS, such as the glass transition temperature, was improved by copper powder, according to the DSC analysis.

The micromechanical characteristics of the micro-composite materials after nanoindentation tests revealed that the indentation hardness and elastic modulus were improved by the addition of copper.

Tribological experiments indicated that 5wt% Cu were responsible for ensuring the lowest values of wear track width and wear volume loss. A severe adhesive wear mode was generated in the case of neat ABS. The mechanism was attenuated into a mild adhesive wear at 5wt% Cu. The hard and sharp asperities in the over-loaded surface of composites as well as the poor adhesion between the filler and the matrix increased the number of wear particles and abraded the surfaces in contact during rotating cycles. The fundamental wear mechanisms of ABS-Cu micro-composites were adhesive and abrasive.

Overall, the unusual combination of improvement in the mechanical, thermal and tribological properties of ABS/Cu micro-composites represented particular results required for industrial-bearing supplies.

## Figures and Tables

**Table 1:** Principal thermal parameters from DSC analysis of ABS and ABS/Cu composites.

Material	Heating Rate (°C/min)	Heat Flow (W/g)	Tg (°C)
ABS Pure	10	−0.339	103.44
5wt% Copper	10	−0.322	106.70
10wt% Copper	10	−0.292	105.15
15wt% Copper	10	−0.259	105.70

## Data Availability

Not applicable.
